# A model of professional self-identity formation in student doctors and dentists: a mixed method study

**DOI:** 10.1186/s12909-015-0365-7

**Published:** 2015-04-29

**Authors:** Pirashanthie Vivekananda-Schmidt, James Crossley, Deborah Murdoch-Eaton

**Affiliations:** Medical Education, University of Sheffield Medical School, Beech Hill Road, Sheffield, S10 2RX UK

**Keywords:** Model, Professional Identity, Medical, Healthcare, Professionalism

## Abstract

**Background:**

Professional self-identity [PSI] can be defined as the degree to which an individual identifies with his or her professional group. Several authors have called for a better understanding of the processes by which healthcare students develop their professional identities, and suggested helpful theoretical frameworks borrowed from the social science and psychology literature. However to our knowledge, there has been little empirical work examining these processes in actual healthcare students, and we are aware of no data driven description of PSI development in healthcare students. Here, we report a data driven model of PSI formation in healthcare students.

**Methods:**

We interviewed 17 student doctors and dentists who had indicated, on a tracking questionnaire, the most substantial changes in their PSI. We analysed their perceptions of the experiences that had influenced their PSI, to develop a descriptive model. Both the primary coder and the secondary coder considered the data without reference to the existing literature; i.e. we used a bottom up approach rather than a top down approach.

**Results:**

The results indicate that two overlapping frames of reference affect PSI formation: the students’ self-perception and their perception of the professional role. They are ‘learning’ both; neither is static. Underpinning those two learning processes, the following key mechanisms operated: [1] When students are allowed to participate in the professional role they learn by trying out their knowledge and skill in the real world and finding out to what extent they work, and by trying to visualise themselves in the role. [2] When others acknowledge students as quasi-professionals they experience transference and may respond with counter-transference by changing to meet expectations or fulfil a prototype. [3] Students may also dry-run their professional role (i.e., independent practice of professional activities) in a safe setting when invited.

**Conclusions:**

Students’ experiences, and their perceptions of those experiences, can be evaluated through a simple model that describes and organises the influences and mechanisms affecting PSI. This empirical model is discussed in the light of prevalent frameworks from the social science and psychology literature.

## Background

Professional self-identity [PSI] can be defined as the extent to which an individual feels like a member of the profession of which they intend to become a part [1,2].

Self-identification is innately subjective. In an observational study of pre-clinical students, Niemi first drew attention to the way in which experiences and the reflection interact to contribute to the individual construction of professional identity [3]. Dornan and colleagues further emphatically highlighted the importance of ‘a state of mind that includes confidence, motivation and a sense of professional identity’ [1]. They interviewed senior students and newly qualified doctors and found that this quality [i.e., the appropriate state of mind] was as important as practical competence in preparing for the world of practice.

Professional identity is a much studied concept in other health professions, especially dentistry; examples include the work of Morison et al. on professional roles and identities which showed that contact with patients and lack of understanding of team roles influenced professional identity within a team [4]. Bebeau et al., have published rich literature linking professional identity formation in dental students to developing ethical clinical practice [5,6].

Given its importance, many authors have called for a better understanding of the processes by which students develop their professional identity. Monrouxe writes that “understanding the process through which we develop our identities has profound implications for medical education…” [7]. Similarly, in a recent paper Cruess et al. argue for a radical change in how we teach and assess professionalism because of the close relationship between developing PSI and developing as a fully-fledged professional [8]. Most agree that medical students arrive at medical school with an identity. Most medical professionalism curricula embrace characteristics, values and beliefs [9]. The accepted view is that these aspects of professionalism influence identity development through a process that involves the renegotiation of the characteristics, norms and values previously held by the person; resulting in the end in the person ‘thinking acting and feeling like a physician’ [10]. The processes of ‘renegotiation’, however, are not well understood.

Several authors have begun to offer answers to the important question of the processes through which PSI is formed. Hafferty argues that in order to develop as a fully fledged professional, the student must understand the profession’s values and norms and that this means they must be supported in obtaining the appropriate opportunities [11]. Weaver and colleagues, interviewing thirteen Australian medical students observed that both professional inclusivity [being ‘treated as future medical professionals’] and social exclusivity [perceptions of themselves as socially separate from non-medical students] were in operation [12]. However, Goldie draws on social psychology to offer the view that the educator’s task is in ‘helping students form, and successfully integrate their professional selves into their multiple identities…’ [13]. In other words, he argues that the process is not so much a transfer or exclusion of identity, but the inclusion of a new identity. Jarvis-Selinger et al.’s paper describes the process of professional identity formation as involving ‘socialization of the person into appropriate roles and forms of participation in the community’s work’ [14]. Cruess et al. advocate a need to better understand this socialization process so that we can engage our students and support them in their ‘determination of the nature of their future professional selves’ [8]. Frost and Regehr critically analysed seminal publications and found evidence of two competing discourses regarding identity formation [15]. They articulate that ‘the discourse of diversity emphasizes individuality, difference, and a plurality of possibilities and advances the notion that heterogeneity is beneficial to medical education and to patients. In contrast, the discourse of standardization strives for homogeneity, sameness, and a limited range of possibilities and conveys that there is a single way to be a competent, professional physician’. The discourse of diversity provides a very different starting point for understanding the development of professional self-identity.

These recent contributions have mainly been theoretical or restricted in their focus. We are not aware of any comprehensive data driven attempt to describe or organise the full range of processes by which healthcare students develop their professional identities. Here, we report a descriptive model of the factors and mechanisms involved in professional identity development. It is derived from the thematic analysis of interview data from a deliberately enriched sample of student doctors and dentists.

## Methods

The study design is an analysis of interview data from a purposive sample of student doctors and dentists who were most likely to have recently experienced a substantial change in their PSI. The themes from the data were utilised to develop a model of PSI formation.

### Recruitment

This study was in compliance with the Helsinki Declaration. We obtained ethical approval for the study from the School of Medicine and Biomedical Sciences Research Ethics Committee of the University of Sheffield [Study number SMBRER47]. All participants were adults and were provided with a study information sheet which specified that participants willingness to take part in the interviews will be taken as consent for fully anonymised data to be used in data analysis and publication. Participants were given the opportunities for questions and clarification prior to participation in the study.

The PSI of student doctors and dentists were tracked using a validated instrument – the PSIQ [2]. The PSIQ asks students to identify their current position between ‘first day student’ and ‘qualified doctor/dentist’ when engaged in each of nine domains of professional activity. Students who were willing to participate in further research were asked to provide their identifying details and indicate their consent to be contacted by the research team. In order to enrich the sample to include as many critical experiences and reflections as possible, we identified the students who had undergone the greatest change in two successive tracking years [two scale points or more]. There is no empirical or statistical significance to two scale points. This enriching approach has been used by other authors [3]. We included both doctors and dentists because we were interested in PSI across health professions and because the two groups experience different curricula and therefore draw upon a wider pool of experiences.

### Interviews

We were keen to include students from all years on the course – particularly the clinical years when students spend a substantial amount of time on clinical attachments. Consequently we made the decision to undertake interviews by telephone, which made it possible to reach students who were remote from the Medical School on clinical attachment. A strength of telephone interviews is that they may reduce social desirability bias because students are less ‘inhibited’ in their responses. One weakness is that the investigator cannot observe or follow up on non-verbal cues.

Semi-structured interviews were used, and conducted by a single investigator [PVS]. Topic questions were developed by discussion within the research meetings of the Academic Unit of Medical Education (Table 1). The questions served as a guide, but the interviewer was free to follow up leads provided during the course of the interview. The interviews were conducted two weeks after the participant had completed the PSIQ for the second time. All interviews were tape recorded and transcribed for analysis.

**Table 1:** **The guide interview schedule**

1.	How do you feel your professional self-identity has changed over the past year?
2.	Have there been any features of the course that have affected this change?
3.	What experiences outside the course have had the greatest effect on this change?
4.	Overall, do you think your training so far as a student has been successful in helping you to be able to professionally self-identify?
5.	Overall, do you feel that by the end of the course, you will be fully prepared to start work as a doctor/dentist? If no, why?
6.	Specific changes:
	Last year you rated yourself [insert value] out of 6 for the question [insert question], compared to this year where you rated yourself [insert value] out of 6 for the same question.
	Can you suggest any explanation for this rise/fall in self-rating?
	[Continue as appropriate]

### Analysis

All interview content was included in the analysis. Data analysis involved coding to reduce and group the data into categories, and data display to identify themes that permit conclusion drawing and verification [16]. The themes arising from the analysis were independently verified by a second investigator and reconsidered and refined as part of ongoing discussions amongst team with the aim of testing and strengthening the credibility of the findings. The investigators analysed the data without considering the existing theoretical literature on the processes of professional identity formation [and, candidly, with a very limited knowledge of that literature]. In other words, the analysis was a bottom up process. Direct quotes are used to support the interpretations as evidence that they are grounded in the data.

## Results

### Recruitment

Sixty-six student doctors and dentists demonstrated a change of two PSIQ scale points or more in two successive tracking years in one or more of the nine domains. There is no empirical significance to a change of two scale points, but it provided a manageable purposively enriched sample size. Of these, forty-six did not provide contact details and/or consent for follow up contact. All twenty remaining students were invited for telephone interview and seventeen of these responded. Eleven of these respondents were student doctors [five males] and six were student dentists [three males]. Participants spanned years two to five in both undergraduate courses.

To provide context for the quotes, we use a five figure code to identify a respondent: M/D = Medical or Dental student, Single figure number = Year of the course, M/F = Male or Female, Double figure number = Unique participant number.

After the student identifying code, we highlight the domains in which the student reported the greatest change. The word[s] identify the domain and the number[s] signify the size of the change [+ is gain, − is fall]. The nine domains of professional activity on the PSIQ are: Teamwork, Communication, Conducting assessments, Cultural awareness, Ethical awareness, Using records, Dealing with emergencies, Reflection & Teaching [2]. Acquiring basic competencies in teaching others is now a requirement of the UK undergraduate medical curricula; it is not uncommon for students to seek additional experience to teach through student societies and extra-curricular activities.

### Themes

The data collected were sufficient for saturation of themes but too small for inspecting gender, course or year-related differences. From the thematic analysis, three themes were identified as representative of the data and encapsulating the experiences behind self-reported changes in professional identity:

1. Opportunities to participate in the professional role [i.e., the common activities that one is expected to undertake as part of their profession]. *Participation* related change was mediated through the mechanisms of *trying out* and *visualisation*.
2. Recognition by others as a quasi-professional. *Recognition* related change was mediated through *counter-transference* and by providing opportunities to *dry-run* for the role of doctor or dentist in informal settings (i.e., independent practice of professional activities when safe to do so).
3. Involvement in extracurricular activities. Extra-curricular activities mainly focussed on teaching and operated through the same mechanisms as above. However there were individual examples of extra-curricular experiences resulting in *self actualisation* and in *changed horizons*. These were only evidenced in the medicine data.

One the most fascinating aspects of the data was the way in which experiences could result either in a change of self-perception or a change in perception about the professional role. There were many examples of PSI falling as a student gained new insights into the professional role and judging that it was more out of reach than they had previously thought, or of PSI rising as a student was able to visualise themselves in the role finding it was less distant than they had feared. Students were *learning about themselves* and *learning about the professional role* for which they were training. Their developing PSI was a function of the overlap between the two.

These themes are expanded and illustrated with quotes below:

### Participation in the professional role

Many students identified that *participation* had a positive influence on their PSI.

*What particularly makes you feel more like a doctor? [Interviewer]*

*“I think the sort of feeling that you are working with people rather than being a student. Feeling able to do lot of the things that they do.....now on your own”. [M5F11: cultural awareness + 2, teaching + 3]*

*“Working in hospitals and being more involved in the treatments given has increased my confidence. Having more responsibility in placements has made me feel that my role is more similar to that of a professional dentist”. [D5M06: Assessing +2, patient records +2, teaching +2]*

For some, these opportunities enabled the student to *try out* or test themselves in the real world of work.

*“I have spent quite a lot of time with F1s [i.e. PGY1 doctors] on placements. That helps you to mentally prepare whereas the course helps you to educationally prepare. For example, meeting a patient with a condition and applying your communication skills and understanding how to take histories…” [M2M02: teaching + 2]*

Those respondents who reported lacking this opportunity, also lacked a sense of capability.

*“This year only 3 lots of 2 hour sessions [with patients] so far and that is why I am less confident…” [M2F01: emergencies-5; records, assessing & cultural awareness-6]*

Through participation other students engaged in *visualisation* – imagining whether they could conceive themselves fulfilling their future professional role. This involved two frames of reference: a growing understanding of the role, and a developing sense of professional self-identity [which may or may not fit the role]. As these frames of reference coalesced some students felt that they were ‘nearly there’.

*“The clinical attachments where you are out there in the real world; the A&E when there is skeleton crew on at night and you are acting as FY1s almost; doing the work as you would. That was really sort of reinstated for us....your knowledge you are putting it into practice and you realise that you are not a million miles away from practice”. [M4F10: teaching + 4, ethics-2]*

For other students [or, in this case, the same student] visualising showed them how far they still had to go.

*“Last year to this year, I realize that a lot is involved in ethical practice and law that you have to be aware of and things like the end of life care has a lot of issues to consider. Last year we hadn’t learnt some of the stuff like the end of life….now I feel I need to know more and consider this carefully”. [M4F10: teaching + 4, ethics-2]*

Students who lacked opportunities to take responsibility frequently reported lacking the information they needed to visualise accurately.

*“I have an idea but actually I don’t know what I’ll be doing as a doctor or as an F1. Seeing this would allow you to prepare yourself and feel like you know what you might be doing. When I get to 4*^*th*^*and 5*^*th*^*year you are going to want to do some of the things F1s do. Just watching means you can’t be sure that you can actually do the task or are capable of doing the task. If you work with the people and do things then you can be confident and capable”. [M2M02: teaching + 2]*

### Being recognised as a quasi-professional

Many of the respondents spoke about how others’ perceptions of them had influenced their self-perceptions.

Healthcare teams’ perceptions of students often took the form of imposing on them the prototype of doctor or dentist [transference]. In some cases this led to *counter transference* [changing self-perceptions to meet others’ expectations].

*“Last year it was kind of more observing but now they are expecting us on our placement to be part of a team and you feel more like a doctor”. [M4F10: teaching + 4, ethics-2]*

*“The teaching we receive during clinics is also very important because the teacher speaks to you as if you are on the same level, not as a student”. [D3F05: Records +2, teaching +2]*

*What particularly makes you feel more like a doctor? [Interviewer]*

*“Being allowed to go near patients and be able to write in the records. Junior doctors encourage you and say it helps you to put your thought in order. They don’t mind you writing in case notes and filing both in psychiatry and neurology. It depends on how much the consultant or junior doctor is happy for you to do that”. [M5F05: teaching + 3, patient records and assessing + 2]*

Outside of the formal curriculum, the perceptions of others were equally important, for example, the expectations of friends and family.

*Has anything else external to the course helped you to feel more like a doctor? [Interviewer]*

*“I have had other people/peers outside the course asking me questions about medical conditions. That has made me feel more like a doctor”. [M2M04: teaching + 3, reflection + 2]*

Some students spoke about undertaking a doctor role outside of any formal healthcare setting in a kind of *dry run*.

*“Friends and family have started to ask my advice on things that they have come down with. I find myself going from being able to tell them nothing about what they have to now where I have an idea of what they’re experiencing”. [M4F08: teaching + 3]*

### Extra-curricular activities

Students frequently cited engagement in teaching activities as relevant to their PSI. This included both formal and informal teaching activities. A range of similar mechanisms operated.

This respondent is saying that they have raised the expectation they have of themselves in response to others’ expectations. This is an example of *counter transference*.

*“Going to schools to give presentations to encourage becoming a donor and so on. That builds your confidence. They look up to you because you are medical student. We have to make the presentation suitable for their level and tailor it for them. I think that is helpful because that make you feel as though you have that identity because you have to make it simple for lay people”. [M4F10: teaching + 4, ethics-2].*

This respondent *tried out* the burden of responsibility in the context of teaching.

*“…to do with the responsibility we had to teach others. The feeling of responsibility that these guys will go away and if you got it wrong the impact on their education will be massive. I took that very seriously. It helped with my professional realisation”. [M4M07: communicating, assessing, teaching + 4; cultural awareness + 3]*

Whilst most comments about extracurricular activities focused on teaching peers and juniors, there were individual examples that suggested other mechanisms. This student describes an experience towards *self-actualisation*:

*“Last year I started a book club via the Students union. For me, coming to this country and starting something like this is big. I thought I should do it because it would make an impact on leadership and teamwork and starting it and having it run successfully has given me confidence in the ward around speaking up”. [M5F05: teaching + 3, patient records and assessing + 2]*

There were also examples of *changed horizons:*

*“Cultural awareness is not just in clinical things....esp. in [this city] it’s reflected through a lot of things that you do. It is not just about the curriculum. Having friends from many different backgrounds and ethnicities on the course”. [M2F01: emergencies-5; records, assessing & cultural awareness-6].*

### Summary

**Figure 1:**
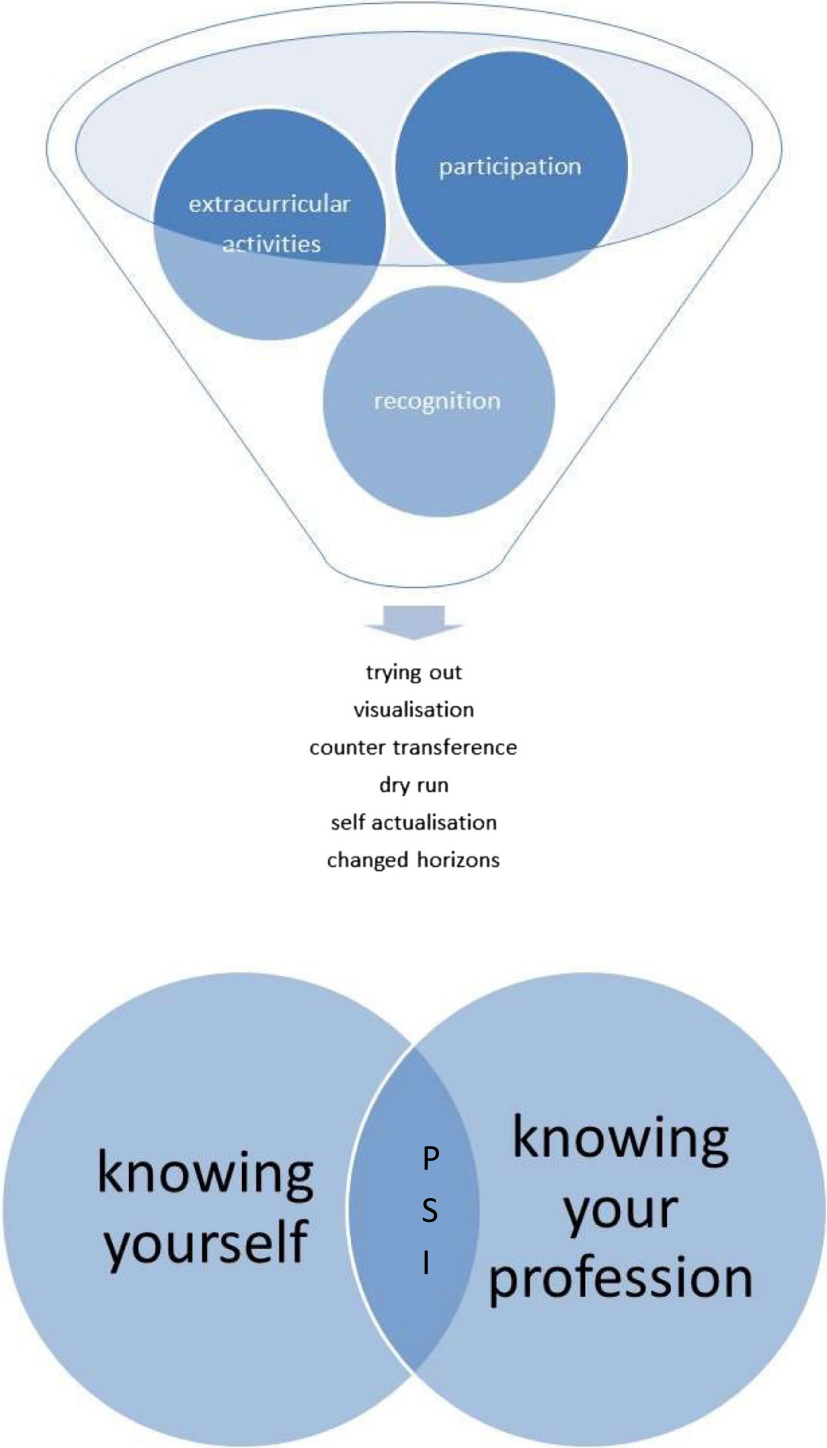
Experiences affecting PSI and the mechanisms mediating change.

Figure 1 presents a model synthesising the observed experiences affecting PSI and mechanisms mediating change.

## Discussion

The analysis and conceptualisation of our model was undertaken without reference to existing theoretical frameworks from the psychological or social sciences literature. However, a number of features of our model align with the themes of that literature, and even add further insights.

First, there is a clear distinction between influential experiences, and the processes by which those experiences are interpreted and constructed. Niemi first drew attention to this important distinction by drawing on the theoretical perspectives of Schon and others [3]. In our data, different respondents report different mechanisms mediating the impact of similar experiences on their PSI. For example, both M2M02 and M4F10 describe working closely with an FY1 on clinical placements – “participating”. However for the former [more junior] student [M2M02], the experience provided an opportunity to *try out* newly acquired skills of history taking and examining in the real world. The latter [more senior] student [M4F10] however was *visualizing* herself doing the FY1 doctor’s job and trying to evaluate if she would be capable.

Second, the social aspects of identification are clearly identified in our data. We have used the terms *transference* and *counter-transference* [in their modern socio-cognitive senses rather than their historical psychotherapeutic senses] [17], because they provide the best description of the way in which self-concept is so profoundly influenced by the implicit and explicit ways in which others identify and treat the student. Susan Andersen and Michele Berk [17], explain that*: “Basic principles of social cognition, and social construct theory in particular, suggest that people … ‘go beyond the information given’about a new person, using an existing social construct’.*

For example both M4F10 and D3F05 refer to implicit expectations of them by others, and the ways that they are spoken to, making them ‘feel’ like a healthcare professional. These are similar to the mechanisms observed by Weaver and colleagues in their paper ‘Part of the Team’ [12]. Indeed, socialisation is one of the most widely studied mechanisms of evolving professional identity with a number of authors describing a dynamic process that evolves in relation to group membership [10,11,13].

Third, the considerable impact of participation is clear from our data. Most respondents, across all years and across both medicine and dentistry cited opportunities to participate – however closely supervised – as pivotal moments. This observation is not new. Jean Lave and Etienne Wenger’s influential book ‘Situated learning: legitimate peripheral participation’ may be said to have fuelled an educational movement [18]. More recently, the unique opportunities and hazards presented by participation for senior students and newly qualified doctors have been described in detail by Dornan and colleagues [1]. Unfortunately, the climate in many Western healthcare systems has made meaningful participation more and more difficult for student doctors and students of some other healthcare professions.

Nevertheless, some key elements of our model help to extend or organising previous work. In particular, the importance of learning the professional role. Our data make it clear that students do not evenly acquire the identity of their chosen health profession. They do not have a settled view of the destination and then self-monitor their progress. PSI continues to develop and change throughout the whole course of a doctor’s training and career. Through the student years, there appears to be an ongoing dialogue addressing two questions with respect to developing as a professional; ‘What is it that I need to become?’ [with reference to the future professional role] and ‘Am I capable of becoming what I need to be?’ [with reference to self]. Many of the experiences that influence their PSI are mediated through the first question. An experience shows a student something more about what they need to become in order to function as a doctor or dentist, and the student is either relieved to discover that she finds herself capable, or concerned that it is out of her reach. We would argue, based on our data, that any analysis of factors affecting the development of PSI is incomplete without this over-arching frame of reference.

Steinert et al.’s call to reconsider how we understand and teach professionalism is relevant here [9]. Our data indicate that role-modelling is important for teaching professionalism but not through a simple ‘copying’ mechanism. Instead, PSI development is a complex process that involves evaluation of group membership, and transference and counter-transference though experiences and interpretation. It is important to consider which aspects of PSI development can be influenced by a professionalism curriculum and whether we are providing our students with an appropriate environment and opportunities to help their negotiation of ‘their future professional selves’ [8]. If we do not carefully consider these issues, we are at the danger of giving our future healthcare professionals ‘surface professionalism’ which ‘ sidesteps issues of identity and treats professionalism as something physicians can put on and take off like one’s stethoscope’ [19].

This study has a number of limitations. Semi-structured interviews by telephone were utilised, and the questions (Figure 1) might be considered narrow in scope. However, as this was a one to one interview, and the conversation was responsive to the agenda of the interviewee rather than driven by the questions, this method nonetheless generated valuable data. The model derived from these interviews is based on a small sample of qualitative data. Hence at this stage, we regard it as a simple descriptive model. We are confident that it describes our data well, but its general application to other situations requires evaluation. The purpose of qualitative data like this is to offer insight into the phenomenon at study rather than offer general conclusions. It is encouraging however that the proposed model aligns with the processes recognised in previous literature.

The most important aspect of our data is that it provides a relatively inclusive or comprehensive model of the experiences and mechanisms affecting PSI development, and expands on aspects not described in any previous work of which we are aware. We have not taken a single theory or mechanism and studied its impact. Rather we have taken a cohort capable of describing a range of experiences and mechanisms and we have used an approach that has not been informed by any particular theory. Consequently our model contributes something unique to the existing literature and, we hope, starts to answer the important question posed by Monrouxe and Weaver [7,12].

## Conclusions

From a research perspective, each of the mechanisms we have identified justifies further exploration.For example, it is important to understand what kinds of participation have the greatest impact, at what stage of a student’s development, and to what extend is the impact dependent on individual differences between students. What kind of support is required to optimise the gain and limit the hazard? How can we support students following potentially challenging experiences, and what is the role of mentors, as opposed to role models, within this? Tim Dornan’s work has already begun to elucidate answers to these questions – highlighting the importance of supported learning as a challenge to the ‘self-direction’ paradigm [1].

Which students stand to gain the most benefit from extra-curricular activities? This is an area that has not been systematically investigated despite waves of enthusiasm and counter-enthusiasm for extra-curricular learning.

What are the hazards of the transference/counter-transference mechanism – especially bearing in mind the diversity/uniformity discourse highlighted by Frost and Regehr [15]. If students engage in counter-transference and may step up to the identity expectations of others, just how damaging are adverse identity expectations? Measures to mitigate the impact of negative stereotypes of the profession, or dumbed-down expectations may prove very important to reduce the extent to which students and junior doctors might ‘step down’ rather than ‘step up’ in response to what is expected of them.

From a curricular perspective the mechanisms we have observed raise an equally fascinating set of possibilities. For example, if students are still learning everyday information about what their professional role involves even at the end of their undergraduate years, it may be that they are spending valuable years of learning too disoriented for optimal learning and development. In this context, interventions that give students an authentic view of the nature of the professional role much earlier in their training are worthy of evaluation. Students typically pursue ‘work experience’ placements before applying to study medicine, often with simplistic and formulaic learning agendas, and usually as an attempt to demonstrate enthusiasm. Rarely are they used as an authentic exploration of the role. Similarly, early clinical placements tend to be dominated by other [competency driven] learning agendas and are usually brief. The challenge is therefore how to embed ‘professional discovery’ placements even before Medical School selection, or at least at the earliest possible opportunity after entry, that will give students an authentic opportunity to grasp what life will be like working as a health professional. Are students’ minds so focussed on success as to make such an intervention impossible?

Can we design the curriculum self-consciously [and transparently] around the stages involved in developing as a health professional so that students can self-evaluate progression by ‘trying out’ , ‘visualising’ , and ‘exploring’ in safety? The concept of a ‘roadmap’ curriculum is not a new idea [20]. One of its attractions is the potential alignment of teachers’ feedback with students’ frames of reference. The variable credibility of feedback and its impact upon PI formation remains one of the great challenges and underexplored areas within medical education [21].
